# A Hepatitis B Virus-Derived Peptide Can Inhibit Infection of Human Lung Cells with SARS-CoV-2 in a Type-1 Interferon-Dependent Manner

**DOI:** 10.3390/v13071227

**Published:** 2021-06-25

**Authors:** Yu-Min Choi, Hyein Jeong, Uni Park, Nam-Hyuk Cho, Bum-Joon Kim

**Affiliations:** 1 Department of Microbiology and Immunology, College of Medicine, Seoul National University, Seoul 110799, Korea; cym486486@snu.ac.kr (Y.-M.C.); zzzzihye@snu.ac.kr (H.J.); woonyi@snu.ac.kr (U.P.); chonh@snu.ac.kr (N.-H.C.); 2 Department of Biomedical Sciences, College of Medicine, Seoul National University, Seoul 03080, Korea; 3 Liver Research Institute, College of Medicine, Seoul National University, Seoul 03080, Korea; 4 Cancer Research Institute, College of Medicine, Seoul National University, Seoul 03080, Korea; 5 Seoul National University Medical Research Center (SNUMRC), Seoul 03080, Korea

**Keywords:** SARS-CoV-2, type I IFN (IFN-I), a hepatitis B virus (HBV)-derived peptide, Poly6, IL-6

## Abstract

The current COVID-19 pandemic has highlighted the urgent need to develop effective therapeutic strategies. We evaluated the in vitro antiviral effect against SARS-CoV-2 of a hepatitis B virus (HBV) hexamer peptide, Poly6, which is capable of eliciting an antiviral effect against human immunodeficiency virus -1 (HIV-1), as a novel HIV-1 integrase inhibitor, and a strong anticancer immune response in an IFN-I-dependent manner, as a novel potential adjuvant in anticancer immunotherapy. Here, we report that Poly6 exerts an anti-SARS-CoV-2 effect, with an estimated 50% inhibitory concentration of 2.617 µM, in the human bronchial epithelial cell line, Calu-3 but not in Vero-E6 cells, which are deficient in type 1 interferon (IFN-I) signaling. We proved via assays based on mRNA profiles, inhibitors, or blocking antibodies that Poly6 can exert an anti-SARS-CoV-2 effect in an IFN-I-dependent manner. We also found that Poly6 inhibits IL-6 production enhanced by SARS-CoV-2 in infected Calu-3 cells at both the transcription and the translation levels, mediated via IL-10 induction in an IFN-I-dependent manner. These results indicate the feasibility of Poly6 as an IFN-I-inducing COVID-19 drug with potent antiviral and anti-inflammatory activities.

## 1. Introduction

Coronaviruses are a diverse group of non-segmented positive-strand RNA viruses with large host range distribution [1]. Coronavirus disease 2019 (COVID-19) is a highly transmissible respiratory disease caused by SARS-CoV-2, a novel severe acute respiratory syndrome coronavirus. Since its first appearance in Wuhan, China, in December 2019, it has spread rapidly worldwide across 223 countries. As of 16 May 2021, there are more than 162 million confirmed cases and over 3.36 million deaths (WHO, COVID pandemic).

Although many drugs are being tested in different clinical trials, none of them are available, and until a sufficient number of people become immune to COVID-19, there is still an enormous risk of contracting the disease, with high potential for mortality and morbidity. Therefore, more efficient and versatile reagents to combat SARS-CoV-2 are urgently needed.

SARS-CoV-2 and SARS-CoV exhibit 79% genome sequence homology, and the structural proteins coded by the genes envelope (E), membrane (M), and nucleocapsid (N), except for the protein coded by the spike (S) gene share over 90% amino acid identity [2]. One of the most pronounced traits that can distinguish SARS-CoV-2 from SARS-CoV is the impaired type I IFN (IFN-I) response induced by SARS-CoV-2 [3]. Interferons (IFNs) are the first-line antiviral cytokines in the innate immune response. Upon recognition of viral infection, IFN-I and IFN-III responses are induced by innate immune sensors. IFN responses induce rapid antiviral mechanisms by activating hundreds of IFN-stimulated genes (ISGs). Recombinant and pegylated IFNs are currently used to treat hepatitis and multiple sclerosis [4,5].

However, the clinical use of IFNs for COVID-19 has been controversial regarding the administration time. While early treatment before the peak of viral replication showed a protective outcome, late administration aggravated the disease [6,7]. Therefore, early administration of IFN may be advantageous for COVID-19 patients [8,9]. Additionally, cytokine inhibitors may help treat the cytokine storm following SARS-CoV-2 infection in severe cases [10].

**Figure 1:**
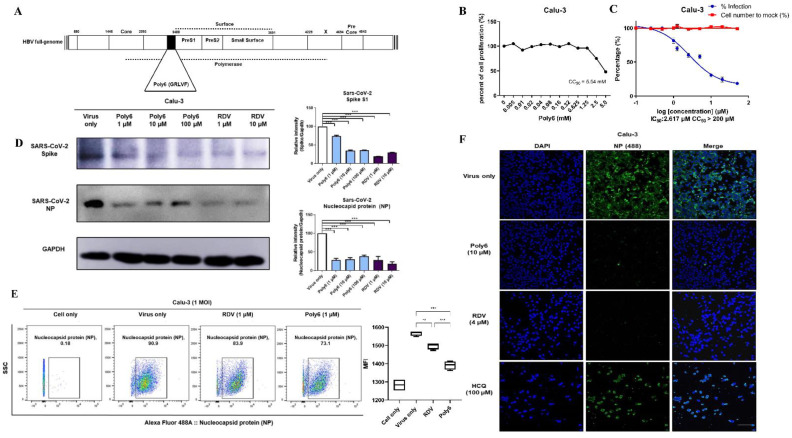
Poly6 inhibits SARS-CoV-2 infection in human lung cell, Calu-3 but not in Vero-E6 cells in a dose-dependent manner. (**A**) Map of Poly6 from the HBV full genome diagram in this study. Poly6 is originated from overlapping HBV polymerase gene regions corresponding to the preS1 deletion region found in patients with severe liver disease. (**B**) Calu-3 cell viability after treatment with different doses of Poly6 was measured using the MTS assay. (**C**) Percent inhibition of SARS-CoV-2 replication and cytotoxicity in Calu-3 cells. Calu-3 cells were infected in triplicate with SARS-CoV-2 at a multiplicity of infection (MOI) of 0.1 in the presence of a range of Poly6 concentrations for 72 h, and the viral replication load was measured using RT-qPCR targeting the RdRp coding region. (**D**) Western blot demonstrating SARS-CoV-2 spike protein and NP following virus infection in Calu-3 cells and inhibitory effects of Poly6 or RDV against SARS-CoV-2 infection. (**E**) Calu-3 cells were infected with SARS-CoV-2 at 1 MOI, and SARS-CoV-2-infected cells were analyzed by flow cytometry. (**F**) Evaluation of the antiviral efficacy of Poly6, RDV, and HCQ in Calu-3 cells via SARS-CoV-2 NP expression immunofluorescence assay. Calu-3 cells were treated with Poly6 (10 µM) and two reference drugs, RDV (4 µM) and HCQ (100 µM), following SARS-CoV-2 infection (0.1 MOI). At 24 hpi, the inhibition of SARS-CoV-2 infectivity by each drug was measured on the basis of the immunofluorescence of the NP. Nuclei are shown in blue (labeled with 40,6-diamidino-2-phenylindole), and NP is shown in green (Alexa 488). Scale bar: 100 μm. The experiments were performed in triplicate and repeated twice for confirmation. ** indicates *p* < 0.01, and *** indicates *p* < 0.001. The results are presented as mean values ± SD. Abbreviations: RDV, remdesivir, HCQ, hydroxychloroquine sulfate.

Previously, we have reported that the 15-, 18-, and 21-nucleotide deletions in the HBV preS1 start region in chronic patients contribute to liver disease progression [11,12,13]. Based on the hypothesis that a 6-mer peptide (Poly6 of the overlapping polymerase corresponding to preS1 deletion could be involved in HBV replication due to the related antiviral responses (Figure 1A we reported that a Poly6 peptide derived from the hepatitis B virus (HBV polymerase could elicit an antiviral effect against human immunodeficiency virus-1 (HIV-1) by suppressing viral integration in infected cells, mainly via inhibition of acetylation of HIV-1 integrase [14]. Moreover, we recently reported that Poly6 could elicit a strong anticancer immune response via TNF/iNOS-producing dendritic cells in an IFN-I-dependent manner in a tumor-bearing mouse model [15]. Therefore, in this study, we sought to evaluate the potential of Poly6 as a novel SARS-CoV-2 inhibitor in the human bronchial epithelial cell line, Calu-3, mainly focusing on enhanced IFN-I production.

## 2. Materials and Methods

### 2.1. Viruses and Cell Cultures

The human lung epithelial cell lines Calu-3 cells and Vero-E6 were maintained at 37 °C with 5% CO_2_ in Dulbecco’s modified Eagle’s medium (WELGENE, Gyeongsan, Korea), 10% fetal bovine serum (FBS; Gibco, Carlsbad, CA, USA), and penicillin and streptomycin (100 U/mL; Gibco, Carlsbad, CA, USA). The SARS-CoV-2 virus (βCoV/KOR/KCDC03/2020) was provided by the National Culture Collection for Pathogens (NCCP), Korea Centers for Disease Control and Prevention (KCDC). All work with the infectious virus was performed in a biosafety level 3 laboratory and approved by the Seoul National University (SNU) and the Korean Disease Control and Prevention Agency (KDCA).

### 2.2. Compounds and Reagents

The Poly6 (GRLVFQ) peptide from the HBV polymerase region overlapping with the preS1 deletion region, mainly discovered from occult HBV infection or severe liver disease progressive patients, was synthesized by the (9-fluorenylmethoxycarbonyl Fmoc)-based solid-phase method and characterized by Peptron Inc. (Cheongju, Korea) (Figure 1A and Figure S1).

Remdesivir (GS-5734, RDV), hydroxychloroquine sulfate (S4430, HCQ), and ruxolitinib (INCB018424) were purchased from Selleck Chemicals (Selleck, Houston, TX, USA). Unless otherwise indicated, all other chemicals were purchased from Sigma-Aldrich (St. Louis, MO, USA).

### 2.3. Infections

Calu-3 cells and Vero E6 cells were plated in 24-well plates (200,000 cells/well). The next day, the cells were infected with SARS-CoV-2 (Calu-3, MOI = 0.1; Vero, MOI = 0.01) for 1 h. After media change, the drugs, including Poly6 and two positive controls, i.e., RDV and HCQ, were added to the culture media. 

### 2.4. Quantification of SARS-CoV-2 Viral RNA Genome Copy Number by RT-qPCR

Cell supernatants were harvested using TRIzol LS reagent (Invitrogen, Carlsbad, CA, USA), and RNA was purified using the chloroform manual method. cDNA was synthesized (Intron), and viral RNA was quantified using the SensiFAST Probe Lo-ROX kit (Bioline, Merdian Biosciences Inc., Memphis, TN, USA). 

### 2.5. Cell Cytotoxicity Assay

Calu-3 and Vero E6 cells were seeded (1 × 10^4^ cells) in 96-well microplates and incubated with increasing concentrations of Poly6 for 24 h. According to the manufacturer’s protocol, cell viability was determined using the MTS assay kit (Promega, Fitchburg, WI, USA).

### 2.6. Immunofluorescence and Dose–Response Curve (DRC) Analysis

Calu-3 cells were seeded and cultivated in two-chamber glass slides (Nunc, Roskilde, Denmark) for 12 h before the experiment. Ten points were assigned for each drug, with concentrations ranging from 0.1 to 50 µM for Poly6 and RDV and from 0.49 to 250 µM for HCQ. The cells were infected with SARS-CoV-2 at a multiplicity of infection (MOI) of 0.1 in the BSL-3 facility. After 24 h, the cells were fixed with 4% PFA and permeabilized with 0.1% Triton-X 100 for 10 min. The cells were stained with primary (1:100, overnight at 4 °C) and secondary (1:1000, 2 h at room temperature) antibodies in 1% BSA in PBS and mounted in a mounting medium containing DAPI (Vectashield, Vetor Laboratories, Inc., Burlingame, CA, USA). Images were captured and analyzed using software to quantify the infection ratio (Leica Software analysis, LAS X and Image J program, version 1.52a), and antiviral activity was measured. DRCs were calculated using Prism7 software (GraphPad, San Diego, CA, USA), with dose–response-inhibition nonlinear analysis. EC_50_ and CC_50_ values were calculated.

### 2.7. IFN-I Neutralization Assay 

For the neutralization assay, Calu-3 cells were seeded in 24-well plates for 12 h. The next day, the cells were pre-incubated with anti-IFNAR1 or isotype IgG antibodies for 2 h. After the cells were washed with PBS, they were infected with SARS-CoV-2 for 1 h, and medium containing PBS (0.5%) or Poly6 (5 or 10 µM) was added for 12–24 h.

### 2.8. Western Blot Analysis

The harvested cells were lysed using RIPA buffer (CST, #9806) containing protease and phosphatase inhibitors (Hoffmann-La Roche Inc., Basel, Switzerland) and incubated for 20 min on ice. The lysed cells were centrifuged for 30 min at 13,000 rpm, and the lysates were collected for western blotting. Protein samples were separated by electrophoresis, transferred to nitrocellulose membranes, and blocked for 1 h with 5% skim milk or bovine serum albumin. The membranes were incubated overnight at 4 °C with the primary antibodies (1:1000). The next day, the membranes were washed with 0.1% Tween-20 in Tris-buffered saline and incubated with horseradish peroxidase-conjugated secondary antibodies (1:2000) for 2 h. After the ECL solution was applied to the membrane, proteins were detected using an imager 

### 2.9. Antibodies

Antibodies to the nucleocapsid (N) protein and spike (S) domains were obtained from Thermo Fisher Scientific (Waltham, MA, USA) and Sino Biological (Wayne, PA, USA). IRF3 (#4962), phospho-IRF3 (#4947S), and STAT1 (#9172) were purchased from Cell Signaling Technology Inc. (Danvers, MA, USA), GAPDH (sc-25778) and phosphor-STAT1 (sc-7988) were purchased from Santa Cruz Biotechnology Inc. (Dallas, TX, USA). Alexa Fluor 488 goat anti-rabbit IgG (H + L) secondary antibody was purchased from Invitrogen (Carlsbad, CA, USA). Vectashield mounting medium with DPAI (H-1500) was purchased from Vector Labs (Burlingame, CA, USA). 

### 2.10. Flow Cytometric Analysis of Nucleocapsid Protein Expression

Calu-3 cells were infected with SARS-CoV-2 (MOI = 1) for 1 h. After washing, fresh medium was added, and PBS, RDV, or Poly6 were added. After 24 h, the cells were detached using PBS containing 1 mM EDTA. After washing, the cells were incubated with polyclonal rabbit anti-SARS-CoV-2 NP antibodies for 1 h on ice, followed by incubation with Alexa Fluor 488-conjugated goat anti-rabbit IgG (1:200) for 1 h. The cells were then analyzed by flow cytometry.

### 2.11. Time-of-Addition Assay

Calu-3 cells were seeded in 24-well plates at a density of 2 × 10^5^ cells per well. For “full-time” treatment, Calu-3 cells were pre-treated with RDV or Poly6 for 1 h and then infected with SARS-CoV-2 for 1 h (0.1 MOI). The virus-containing media were then removed, and the cells were incubated with drug-containing media until the end of the experiment. For the “entry” treatment, the drugs were added to the cells for 1 h before infection, and after infection, the medium containing virus and drug was removed, and fresh medium was added; the cells were incubated until the end of the assay. For the “post-entry” treatment, the drugs were added to the cells after infection, and incubation lasted until the end of the assay.

### 2.12. qPCR Array Analysis

Total RNA was extracted from SARS-CoV-2 infected Calu-3 cells, and cDNA was synthesized. Specific gene expression was analyzed using the AccuTarget qPCR Screening kit (Bioneer, Daejeon, Korea) and qPCR array service.

### 2.13. Enzyme-Linked Immunosorbent Assay (ELISA)

To determine cytokine production, the cell supernatants were harvested 48 h post-infection, and IL-6 levels were detected using human IL-6 uncoated ELISA (Invitrogen, Carlsbad, CA, USA).

### 2.14. Plaque Reduction Assay in Vero-E6 Cells

The cells were infected with 80 PFU of SARS-CoV-2 for 1 h at 37 ℃. After aspiration and washing, the cells were overlaid with medium containing 0.8% agarose in DMEM and the antiviral agents and incubated for 72 h. The wells were fixed with 4% paraformaldehyde. After removing the agarose, the cells were stained with primary SARS-CoV-2 NP antibody and alkaline phosphatase (AP)-conjugated secondary antibody, and the signal was developed using NBT/BCIP for plaque count.

### 2.15. Quantification and Statistical Analysis

Data analyses were performed using the Prism 7 software (GraphPad Software, San Diego, CA, USA). Data are presented as mean ± SD. Statistically significant differences were determined by one-way analysis of variance. The *p*-value of statistical significance was set at *p* < 0.05 (*), 0.01 (**), or 0.001 (***).

## 3. Results

### 3.1. Poly6 Inhibits SARS-CoV-2 Infection in Calu-3 but Not in the Vero-E6 Cell Line in a Dose-Dependent Manner

To determine whether Poly6 (Figure 1A) has antiviral activity against SARS-CoV-2, Calu-3, human bronchial epithelial cells, and Vero E6, African green monkey kidney cells were used. First, cytotoxicity in Calu-3 and Vero E6 cells was determined using the MTS assay. The cells were treated with various concentrations of Poly6 for 24 h at 37 °C, and their viability was measured. CC50 of the Poly6 in both cell lines was significantly high (5.54 mM), indicating the safety of Poly6 even at high concentrations (Figure 1B and Figure S2).

Next, to test the direct inhibition capability of Poly6 against SARS-CoV-2, Calu-3 cells were infected at 0.1 MOI and treated with Poly6. The culture supernatant was harvested at 72 h, and RT-qPCR for viral RNA measurement was performed. We found that Poly6 treatment showed inhibitory efficacy, with an IC50 value of 2.617 µM (Figure 1C). To further investigate the antiviral effect of Poly6 against SARS-CoV-2 at the translational level, we performed western blot assays to detect the NP and spike proteins of SARS-CoV-2. Our western blot data consistently showed that Poly6 has antiviral activity against SARS-CoV-2, almost comparable to that of a potent reference drug, RDV (Figure 1D). The infection of Calu-3 cells and the anti-viral effects of Poly6 and RDV were further monitored by staining the cells with an antibody against the SARS-CoV-2 NP, analyzed by flow cytometry. The cells were infected with the virus for 1 h at 1 MOI, and the drug- or PBS-treated cells were incubated for 24 h. As shown in Figure 1E, infected cells at a high MOI (=1) yielded infection rates as high as approximately 90%, and treatment of the cells with RDV or Poly6 reduced the percentage of infected cells by approximately 20%. Furthermore, the SARS-CoV-2 NP-based immunofluorescence assay also showed a potent inhibitory effect of Poly6 against SARS-CoV-2 infection in Calu-3 cells (Figure 1F).

### 3.2. Immunofluorescence and Dose–Response Curve Analysis Showed a Potent Inhibitory Effect of Poly6 against Nucleocapsid Protein (NP)

**Figure 2:**
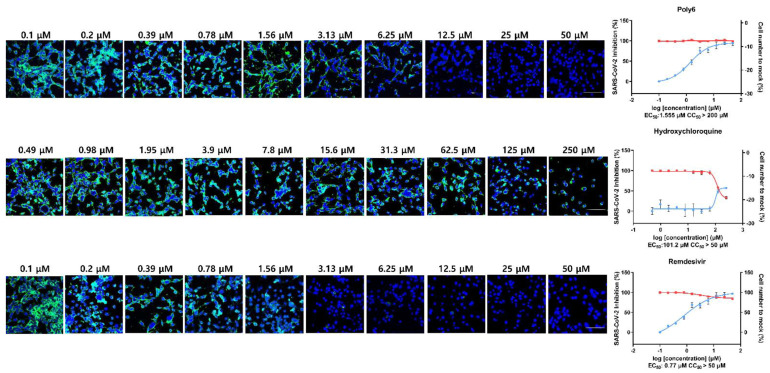
Immunofluorescence and dose–response curve analysis of Poly6 and reference drugs. Poly6 and two reference drugs, remdesivir (RDV) and hydroxychloroquine sulfate (HCQ), were serially diluted to 10 concentrations ranging from 0.1 to 50 µM for Poly6 and RDV and 0.47 to 250 µM for HCQ. SARS-CoV-2 infectivity and the inhibition capacity of each drug were measured by immunofluorescence of NP. EC50 and CC50 are indicated under each graph. Nuclei are shown in blue, and NP is shown in green (Alexa 488). Graphs on the right panel of the images: the blue line shows the percent infectivity of SARS-CoV-2 in treated cells compared to the control group, and the red line indicates cell number in treated cells compared to mock-treated cells in percentage (%). Scale bars: 100 μm.

For the analysis of the drugs’ dose–response curve (DRC), Calu-3 cells were treated with drugs including Poly6, Remdesivir, and Hydrochloroquinesulfate after infection with SARS-CoV-2. Calu-3 cells were treated with the drugs in 10 serially diluted concentrations, and the SARS-CoV-2 infectivity and inhibition capacity of each drug were analyzed. Poly6 showed a potent inhibitory effect in immunofluorescence analysis (IFA), low EC_50_ (1.555 µM), and a significantly high CC_50_ value (>200 µM). RDV showed very low EC_50_ (0.77 µM) with moderate CC_50_ (>50 µM), whereas HCQ showed marginal inhibitory effects in Calu-3 cells, with high EC_50_ (101.2 µM) and moderate CC_50_ (>50 µM), which is consistent with the results of previous studies (Figure 2) [16].

### 3.3. Poly6 Displayed Limited Ability to Control SARS-CoV-2 in Vero E6 Cells

**Figure 3:**
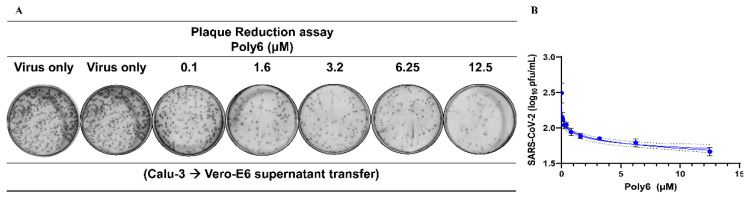
Infection of Calu-3 cells and virus quantification by the plaque assay in Vero-E6 cells. Calu-3 cells were infected with SARS-CoV-2 (MOI = 1). At 40 hpi, the supernatant was collected, and the virus quantified by the plaque assay in Vero-E6. (**A**) Plaque reduction assay. (**B**) Graph shows dose–response curve of Poly6 antiviral activity using 1 MOI virus inoculum. Values are mean ± SD of duplicate readings.

Meanwhile, Poly6 rarely inhibited SARS-CoV-2 infection in Vero E6 cells (Figure S2), which are deficient in IFN-I signaling. While the inhibitory antiviral effect of Poly6 in Calu-3 cells was not observed in Vero-E6 in either RT-qPCR or plaque assays, RDV and HCQ showed significant antiviral activity. On the contrary, when we infected Calu-3 cells with SARS-CoV-2 (1 MOI) and performed virus quantification by the plaque assay in Vero-E6 cells, Poly6 efficiently blocked SARS-CoV-2 infection, and the EC50 calculated in Vero-E6 cells was 1.51 µM (Figure 3 and Figure S2D). Therefore, we found that Poly6 exerts a potent antiviral effect in human lung epithelial cells Calu-3, but not in Vero-E6.

### 3.4. Poly6 Exerts an Anti-SARS-CoV-2 Effect in an IFN-I-Dependent Manner in Calu-3 Cells

**Figure 4:**
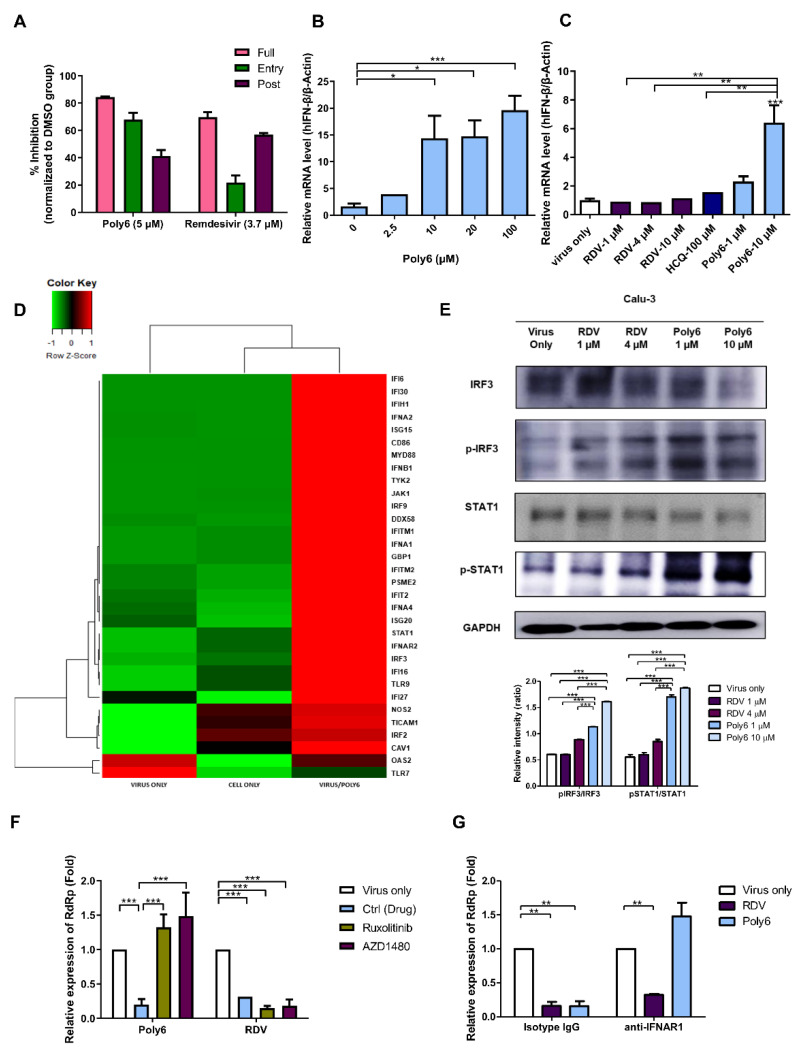
Poly6 exerts an anti-SARS-CoV-2 effect in Calu-3 cells in an IFN-I-dependent manner (**A**) Time-of-addition experiment of Poly6 and RDV and three time points of administration were tested. (**B**) Calu-3 cells were treated with Poly6 in the absence of infection, and the mRNA expression level of IFNβ was evaluated via RT-qPCR (**C**). Calu-3 cells were treated with RDV, HCQ, and Poly6 following infection, and the mRNA expression level of IFNβ was evaluated via RT-qPCR. (**D**) mRNA expression profiles for IFN-I response genes encoding interferons/receptors in SARS-CoV-2 infected Calu-3 cells and following treatment of Poly6. A heatmap is depicted. (**E**) Western blot analysis of Calu-3 cells treated with PBS, RDV, and Poly6 following SARS-CoV-2 infection. (**F**) The IFN-I-dependent antiviral effect of Poly6 against SARS-CoV-2 was investigated with Ruxolitinib and AZD1480. (**G**) Calu-3 cells were pre-incubated with IFNAR1 and isotype IgG antibodies (10 µg/mL) for 2 h and treated with RDV or Poly6 following SARS-CoV-2 infection. RT-qPCR measured SARS-CoV-2 RdRp expression level. Data represent the mean ± SD. * *p* < 0.05, ** *p* < 0.01, and *** *p* < 0.001. The experiments were performed in triplicate and repeated twice for confirmation. Abbreviation: RdRp, RNA-dependent RNA polymerase.

To investigate the antiviral mechanism underlying the effect of Poly6 against SARS-CoV-2, we performed a time-of-addition assay in Calu-3 cells. The cells were incubated with Poly6 during the virus entry stage (entry), post-entry process (post-entry), or the entire time of infection (full time), and virus production at 24 hpi was quantified using RT-qPCR. As shown in Figure 4A, Poly6 acted on both the early and the post-entry stages of the replication process but showed a more substantial influence on the early step (~68% inhibition), with a lesser influence on the post-entry stages (~41% inhibition), suggesting that Poly6 could play an essential role in inhibiting the early stages of viral replication. 

Then, we performed a competitive binding assays by Spike-ACE2 binding ELISA, and serial dilutions of Poly6 were added for competitive binding to SARS-CoV-2 RBD. However, as shown in Figure S3A, Poly6 did not show an inhibitory effect of Spike–ACE2 binding. Meanwhile, as ACE2 has been known to be an interferon-stimulated gene (ISG), the role and clinical outcomes of IFNs in SARS-CoV-2 infection condition are controversial. A recent study revealed that deltaACE2, the non-functional and transcriptionally independent truncated isoform of ACE2, is a type of ISG but not an ACE2; therefore, induction of dACE2 upon IFN treatment is independent of the entry of SARS-COV-2 [17]. Consistently, our results showed that transcription of deltaACE2 as well as ISG induction, such as IFIT1 and Mx1 was also increased following treatment with Poly6, but not ACE transcription level (Figure S3B). These results suggested that the antiviral effects of Poly6 against SARS-CoV-2 could be associated with IFN-I signaling.

Recently, we have reported that Poly6 can lead to TNF/iNOS-producing (Tip) DC, mediating an anticancer effect via enhanced IFN-I in vaccinated mice [15]. Therefore, we further investigated the antiviral mechanism of Poly6, focusing on IFN-I as a potential antiviral signaling molecule. Consistent with our previous study, Poly6 strongly increased the mRNA level of human IFN-β in Calu-3 cells in the absence of SARS-CoV-2 infection (Figure 4B). Next, IFN-I signaling induced by Poly6 was investigated following SARS-CoV-2 infection. We found that Poly6 dramatically increased the transcription level of human IFN-β genes in a dose-dependent manner compared to RDV and HCQ (Figure 4C). Furthermore, we sought to check the transcriptional changes in IFN-I-related genes following treatment with Poly6. To this end, we analyzed the transcriptional profiles of SARS-CoV-2-infected cells and infected cells subjected to treatment with Poly6 (12 hpi). We found that transcription of more than 40 IFN-I-related genes, including IFIH1, IFNb1, IFNA1, IFNA4, IRF3, IRF9, TYK2, IFIT2, STAT1, IFI16, JAK1, and IFI6, was upregulated (Figure 4D and Figure S4A). We then measured the protein expression levels of two critical genes related to IFN-I signaling, i.e., IRF3 and STAT1. Treatment with Poly6 following SARS-CoV-2 infection significantly increased the levels of phosphorylated IRF3 and phosphorylated STAT-1, suggesting that the antiviral effect of Poly6 against SARS-COV-2 could be exerted via the IRF3–IFN-I axis (Figure 4E).

To further clarify the IFN-I-dependent anti-viral effects of Poly6, two IFN-I signaling inhibitors were used. Calu-3 cells were pre-treated with ruxolitinib, targeting JAK/STAT signaling induced by IFN-I or III, and AZD1480, a potent JAK1 inhibitor, and Poly6 or RDV were treated, following SARS-CoV-2 infection. We found that ruxolitinib and AZD1480 treatments significantly reversed the reduced virus replication level induced by Poly6 treatment, but not in the RDV group (Figure 4F). We also assayed the anti-SARS-CoV-2 effect of Poly6 after treatment with an IFN receptor-neutralizing antibody (IFNAR1). As expected, the antiviral effects of Poly6 against SARS-CoV-2 were also recovered in the IFNAR1-blocking group (Figure 4G). Together, our data indicate that Poly6 exerts an anti-SARS-CoV-2 effect in an IFN-I-dependent manner in Calu-3 cells.

### 3.5. Poly6 Inhibits Pro-Inflammatory Cytokine Production in SARS-CoV-2-Infected Calu-3 Cells in an IFN-I-Dependent Manner

**Figure 5:**
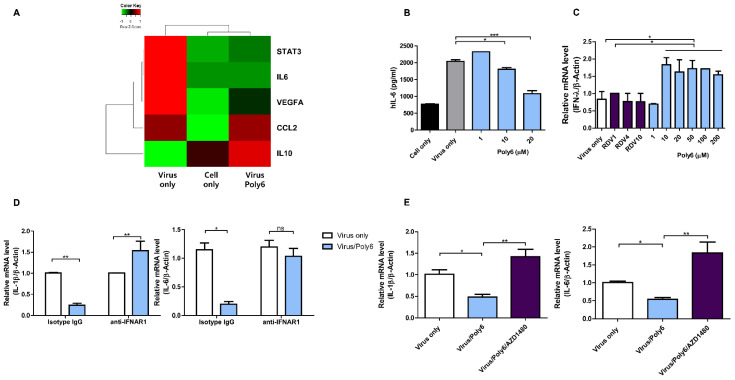
Poly6 inhibits pro-inflammatory cytokine production in SARS-CoV-2-infected Calu-3 cells. (**A**) A heatmap of STAT3, IL-6, VEGFA, CCL2, and IL-10 was constructed using the qPCR array in Calu-3 cells infected with SARS-CoV-2 and treated with Poly6. The colors show fold changes as ddCt normalized by an endogenous control (GAPDH) and comparing Virus-only or Poly6 groups to uninfected cells. (**B**) The IL-6 cytokine immunoassay was performed with human IL-6 ELISA. (**C**) IFN-λ mRNA expression levels in Calu-3 cells treated with PBS, RDV, or Poly6 following SARS-CoV-2 infection. (**D**,**E**) Calu-3 cells were pre-incubated with isotype IgG or IFNAR1 antibodies (10 µg/mL) for 2 h at 37 °C (**D**). Calu-3 cells were pre-treated with AZD1480 for 2 h at 37 °C (**E**). After SARS-CoV-2 infection, PBS or Poly6 was added for 24 h. Cells were collected, and RT-qPCR was performed to analyze the mRNA levels of IL-1β and IL-6. Genes were normalized to β-actin, and data are shown as fold changes. The experiments were performed in triplicate and repeated twice for confirmation. Data represent the mean ± SD and * *p* < 0.05, ** *p* < 0.01, *** *p* < 0.001, ns: not significant.

SARS-CoV-2 is known to induce the production of significant amounts of pro-inflammatory cytokines and mediators, such as IL-1, IL-6, and vascular endothelial growth factor (VEGF), which results in a cytokine storm in severe COVID-19 cases [18,19,20]. Therefore, significant efforts are currently underway to identify pro-inflammatory cytokines in severe cases [21,22,23]. Several studies have reported the usefulness of anti-inflammatory drugs in patients with severe COVID-19 [24,25]. In our transcription profile assay, treatment with Poly6 downregulated pro-inflammatory genes encoding cytokines and chemokines such as IL-6 (and relevant STAT3) and VEGFA, which were highly upregulated following SARS-CoV-2 infection (Figure 5A). To check whether Poly6 alleviates elevated IL-6 production induced by SARS-CoV-2 infection at the translational level, we measured the amount of IL-6 in the supernatant collected from Calu-3 cells infected with SARS-CoV-2 and treated them with Poly6. Accordingly, IL-6 levels were highly elevated by infection with SARS-CoV-2 but mitigated to the average cell level after treatment with Poly6 in a dose-dependent manner (Figure 5B).

We hypothesized that the anti-inflammatory activity of Poly6 was also derived from IFN-I signaling because IFN-I is known for its antiviral activity and anti-inflammatory effects [26,27,28]. IFN-β can induce potent anti-inflammatory cytokines, typically, IL-10 and other mediators such as suppressor of cytokine signaling protein, SOCS-1, and finally reduce pro-inflammatory cytokines including IL-6, IL-1β, and other inflammatory mediators [26,29,30]. Our transcription profile assay showed upregulation of STAT1, IRF3, IRF9, JAK1, IFI16 (Figure 4D and Figure S4A), and, ultimately, IL-10, with downregulation of pro-inflammatory genes such as IL6, VEGFA, and IL-1β in Poly6-treated cells (Figure 5A and Figure S4B).

To determine whether the anti-inflammatory activity of Poly6 was dependent on IFN-I signaling, we measured the gene expression levels of IL-6 and IL-1β after blocking IFN signals using an IFN receptor-neutralizing antibody (IFNAR1) or AZD1480. As shown in Figure 5D,E, the downregulated IL-6 and IL-1β mRNA levels in Poly6-treated cells were recovered in IFNAR1- or AZD1480-treated cells, suggesting an IFN-I dependence of Poly6’s anti-inflammatory activity. Furthermore, treatment with Poly6 following SARS-CoV-2 infection also upregulated IFN-λ (Figure 5C), which shares the JAK–STAT pathway with IFN-I and can induce similar antiviral responses.

Together, our data indicate that Poly6 inhibits pro-inflammatory cytokine production in SARS-CoV-2-infected Calu-3 cells in an IFN-I-dependent manner, suggesting its potential as an IFN-I-inducing drug for COVID-19 treatment, exerting dual antiviral and anti-inflammatory activities.

## 4. Discussion

Since the COVID-19 outbreak in January 2020, many investigations and clinical trials have been initiated to identify therapies capable of suppressing disease severity [31,32,33]. Among the clinical trials authorized worldwide, more than 50 trials are based on IFN-I signaling, including the use of IFN-α and IFN-β alone or in combination therapy at various dosages and by various delivery routes [34,35,36]. In general, IFN-I can exert antiviral activity via two actions: one involves its paracrine direct viral killing, and the other the development of neutralizing antibodies and virus-specific T cells [37,38]. Multiple studies supporting that IFN-I could play a pivotal protective role in SARS-CoV-2 infection have recently been reported [7,39,40].

In this study, we sought to explore whether Poly6 can also elicit an antiviral effect against SARS-CoV-2 infection in Vero-E6 cells defective in IFN-I production. In contrast to the two reference drugs, RDV and HCQ, we failed to find evidence of the anti-SARS-CoV-2 effect of Poly6 in the Vero-E6 system by RT-qPCR and plaque assay (Figure S2), indicating that the anti-SARS-CoV-2 effect of Poly6 is IFN-I dependent. Of note, we also found that the Poly-6-induced antiviral effect was recovered when inhibitors interfering with IFN-I signaling or blocking Abs were administered, further proving its dependence on IFN-I signaling (Figure 4F-G).

The long-lasting challenge of exogenous IFN-I in patients can evoke several side effects [41,42]. Instead, Poly6, as a novel IFN-I inducer composed of only six amino acids, could minimize the risk of potential side effects generated by exogenous IFN-I and provide some advantages in combinatorial treatment with other drugs over exogenous IFN-I.

Paradoxically, in addition to its antiviral effect, IFN-I, particularly IFN-β, can also exert anti-inflammatory effects by enhancing the production of immunosuppressive cytokine IL-10, enhancing the expression of SOCS-1, downregulating matrix metalloproteinase–9 (MMP-9), or inducing IFI16-mediated inflammasome inhibition [26] (graphical abstract). Therefore, IFN-Is have been widely applied to treat autoimmune and inflammatory diseases, such as multiple sclerosis (MS) [5], familial Mediterranean fever (FMF) [43], and Behcet’s syndrome [44]. In this study, our transcription profile assay indicated that Poly6 treatment of SARS-CoV-2-infected Calu-3 cells led to the upregulation of the genes *STAT1*, *IRF3*, *IRF9*, *JAK1*, and *IFI16* (Figure 4D and Figure S4) and of two immunosuppressive molecules, IL-10 and SOCS-1, but to the downregulation of pro-inflammatory genes such as *IL-6* and *IL-1β*. We then confirmed that the downregulation of IL-6 production or transcription of IL-6 and IL-1β induced by Poly6 was recovered by treatment with an IFN-I inhibitor or blocking Abs, due to further blocked type I IFN signals, which are regulators of IL-1 production [45], suggesting that the anti-inflammatory effect of Poly6 is also dependent on IFN-I signaling.

In conclusion, our data indicate that a 6-mer HBV-derived peptide, Poly6, exerts a potent ani-SARS-CoV-2 effect and exerts anti-inflammatory effects in virus-infected human lung cells Calu-3, in an IFN-I-dependent manner. These results suggest the feasibility of Poly6 as a new IFN-I-inducing drug for COVID-19 treatment, with potent antiviral and anti-inflammatory activities.

## 5. Conclusions

IFN-I signaling plays very pivotal role in the control of SARS-CoV-2 infection. We demonstrated that a hepatitis B virus-derived peptide, Poly6, has a potent antiviral activity against SARS-CoV-2 infection of the human lung cells Calu-3 in an IFN-I-dependent manner. Moreover, it reduced IL-6-mediated inflammation in infected Calu-3 cells. These results indicate the potential of the hepatitis B virus (HBV) 6-mer peptide Poly6 as a drug for COVID-19 treatment, with potent antiviral and anti-inflammatory activities mediated by IFN-I signaling.

## Data Availability

The data presented in this study are available on request from the corresponding author.

## Supplementary Materials

The following are available online at https://www.mdpi.com/article/10.3390/v13071227/s1, Figure S1: The Poly6 (GRLVFQ) peptide was synthesized by the Fmoc (9-fluorenylmethoxycarbonyl)-based solid-phase method. The area percent report and MS data report was performed, Figure S2: Poly6 displayed limited ability to control SARS-CoV-2 in Vero E6 cells, Figure S3: Poly6 and Remdesivir tested for neutralizing activity with SARS-CoV2 Spike-ACE2 binding assay, Figure S4: mRNA expression profiles for IFN-I response genes encoding interferons/receptors in SARS-CoV-2 infected Calu-3 cells and following treatment of Poly6 Figures S5–S13: The whole Western blot figures.
