# Supplementary material for: A Hepatitis B Virus-Derived Peptide Can Inhibit Infection of Human Lung Cells with SARS-CoV-2 in a Type-1 Interferon-Dependent Manner
Source: Viruses. 2021 Jun 25;13(7):1227. doi: 10.3390/v13071227 (PMC8310041; doi:10.3390/v13071227)
Supplement: Supplementary file 1 [file viruses-13-01227-s001.zip › viruses-1243991-supplementary.pdf]

SUPPLEMENTARY LEGENDS

Figure S1.

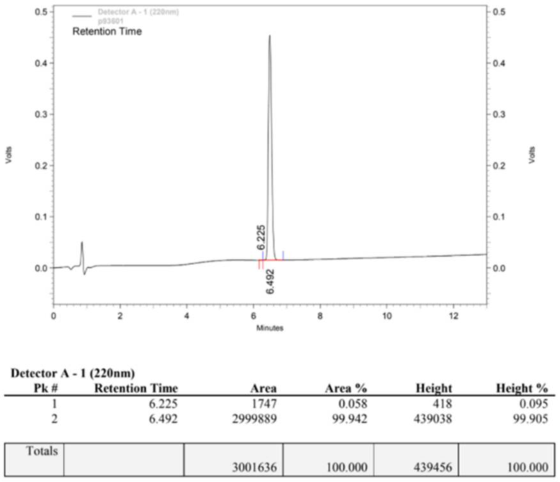

(A) Area % Report

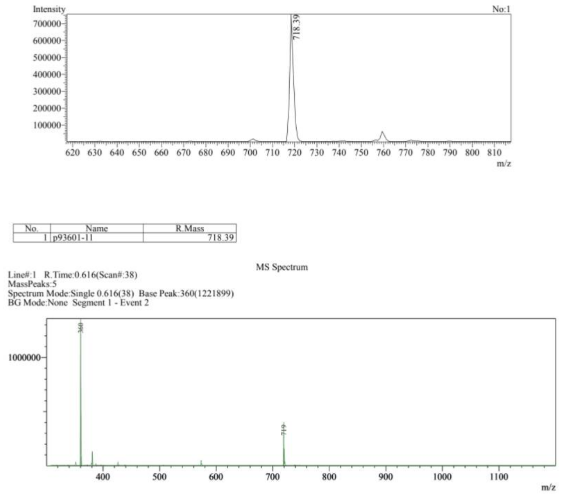

(B) MS Data Report

The Poly6 (GRLVFQ) peptide was synthesized by the Fmoc (9-fluorenylmethoxycarbonyl)-based solid-phase method. The area percent report and MS data report was performed.

Figure S2.

(A)

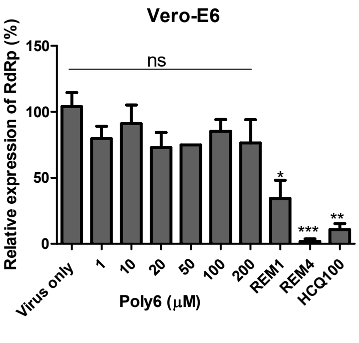

(B)

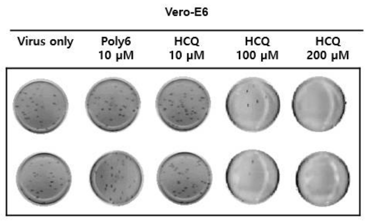

(C)

Poly6 displayed limited ability to control SARS-CoV-2 in Vero E6 cells (A) Vero-E6 cell viability subject to different doses of Poly6 treatment was measured by MTS assay. **(B)** SARS-CoV-2 viral load reduction assay in Vero-E6 cells. **(C)** SARS-CoV-2 plaque reduction assay in Vero-E6 cells. (D) Plaque assay of Poly6 on Calu-3 cells (Calu-3→Vero-E6 supernatant transfer). The experiments were performed in triplicate and repeated twice for confirmation. *indicates p <0.05, **indicates p<0.01, and ***indicates p<0.001. The results are presented as mean values±SD. Abbreviation: RDV, remdesivir.

HCQ, hydroxychloroquine sulfate.

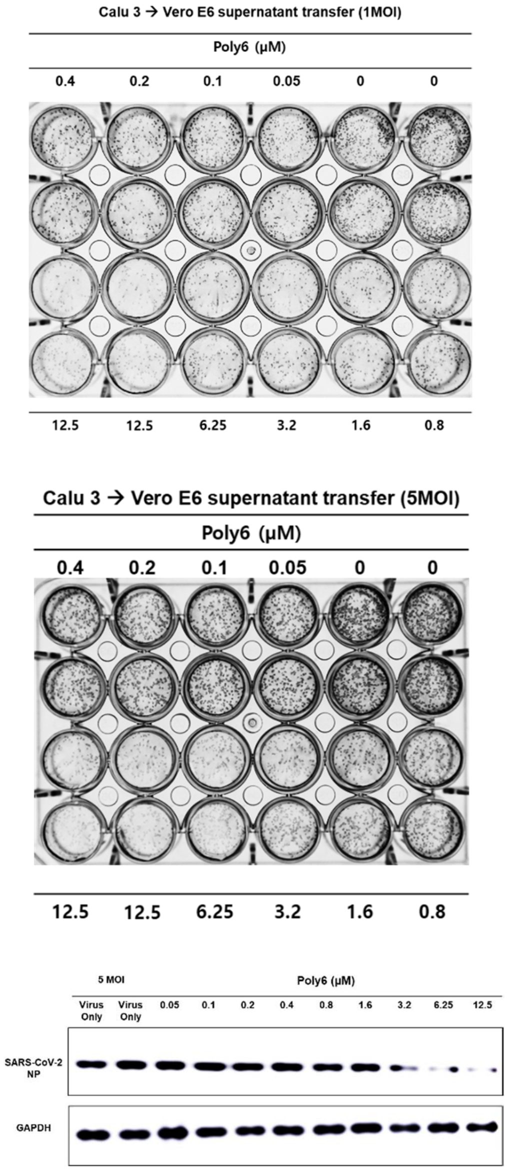

(D)

Figure S3.

(A)

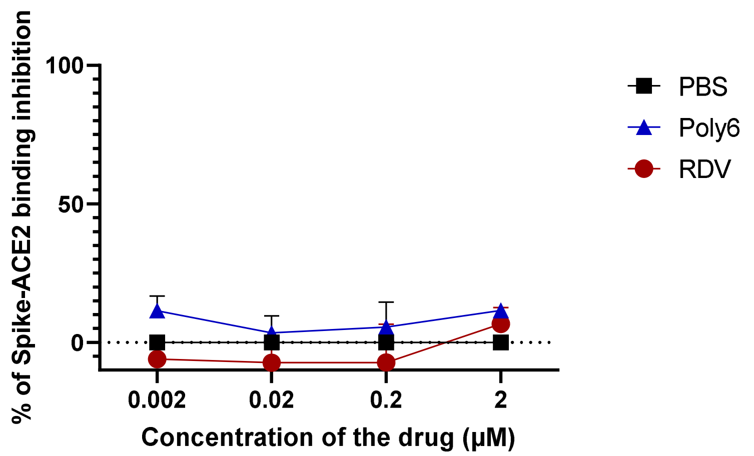

(B)

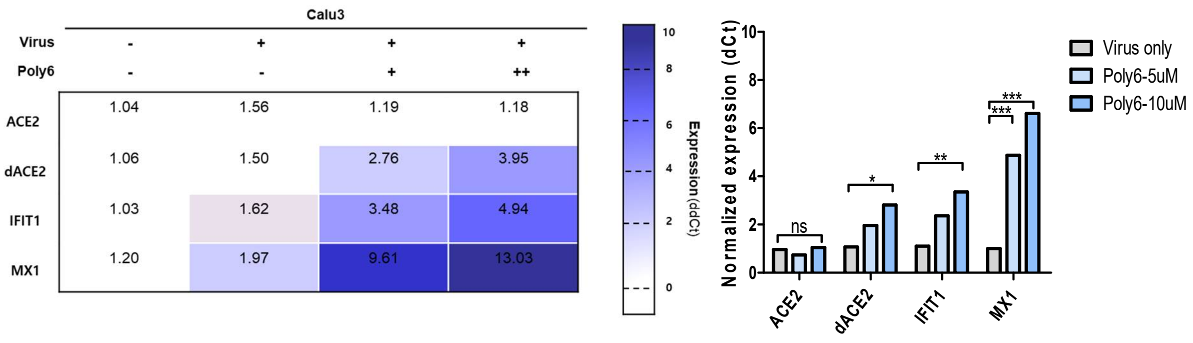

Poly6 and Remdesivir tested for neutralizing activity with SARS-CoV2 Spike-ACE2 binding assay

(A) Competitive binding assay by Spike-ACE2 binding assay ELISA. SARS-CoV-2 RBD protein was coated on 96-well plates; recombinant ACE2 and serial dilutions of Poly6 and RDV were added for competitive binding to SARS-CoV-2 RBD. Values are the mean±SD. Experiments were performed in duplicate. (B) The expression of ACE2, dACE2, and positive control ISGs, IFIT1 and MX1 in Calu-3 cells treated with Poly6 following SARS-CoV-2 infection. A heatmap was depicted and the colors represent fold change levels as ddCt normalized by control (bActin). Data represent the mean±SD. $*p < 0.05$, $**p < 0.01$, and $***p < 0.001$. The experiments were performed in triplicate and repeated twice for confirmation.

Figure S4.

(A)

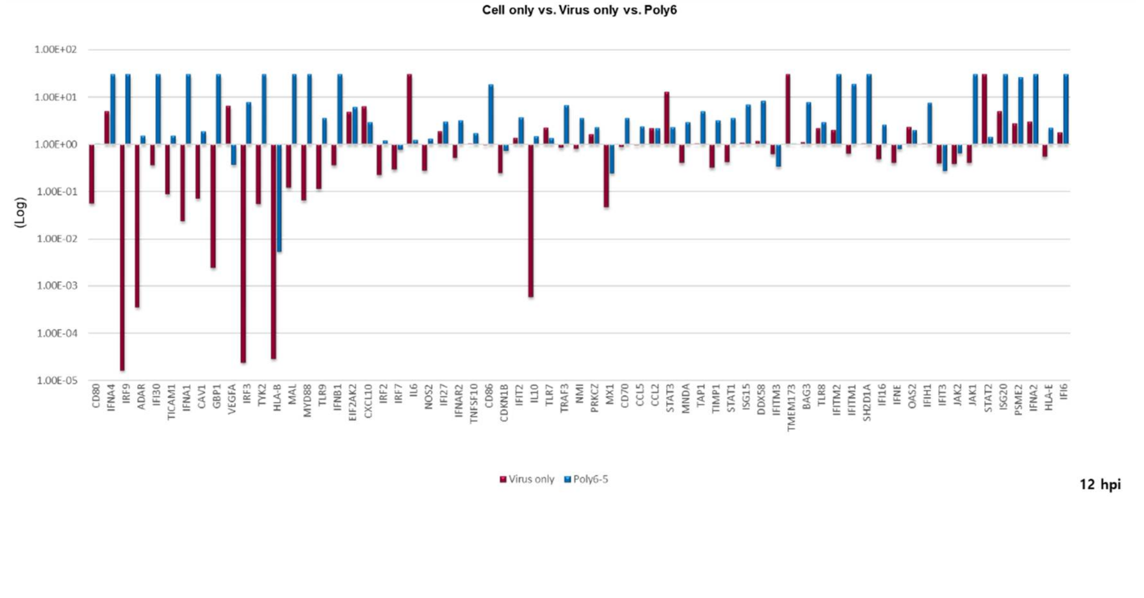

(B)

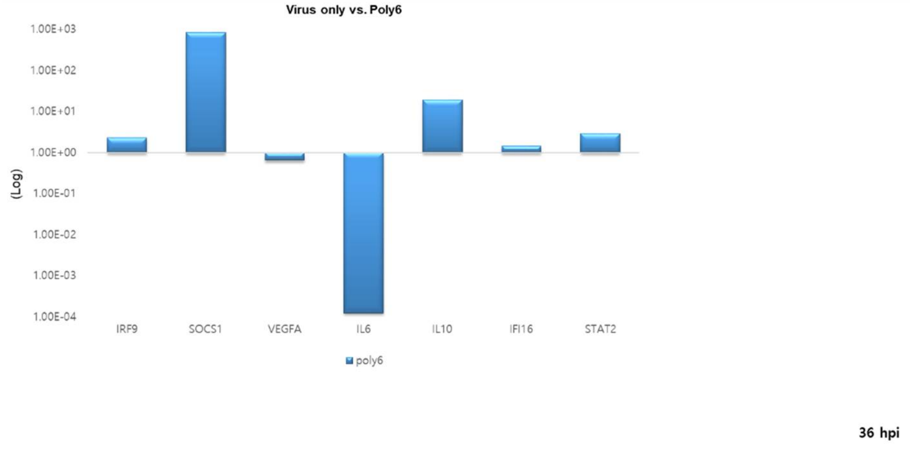

mRNA expression profiles for IFN-I response genes encoding interferons/receptors in SARS-CoV-2 infected Calu-3 cells and following treatment of Poly6. (A) mRNA expression profiles in SARS-CoV-2 infected cells and treatment of Poly6 (12hpi). Fold changes in Virus only or Poly6-treated group were normalized to Cell only group **(B)** mRNA expression profiles in SARS-CoV-2 infected cells and treatment of Poly6 (36hpi). Fold changes in the Poly6-treated group were normalized to Virus only group. The experiments were repeated twice for confirmation. The experiments were performed in duplicate and repeated twice for confirmation.

Western blot whole blot data

Figure S5.

SARS-CoV-2
Spike (S1)

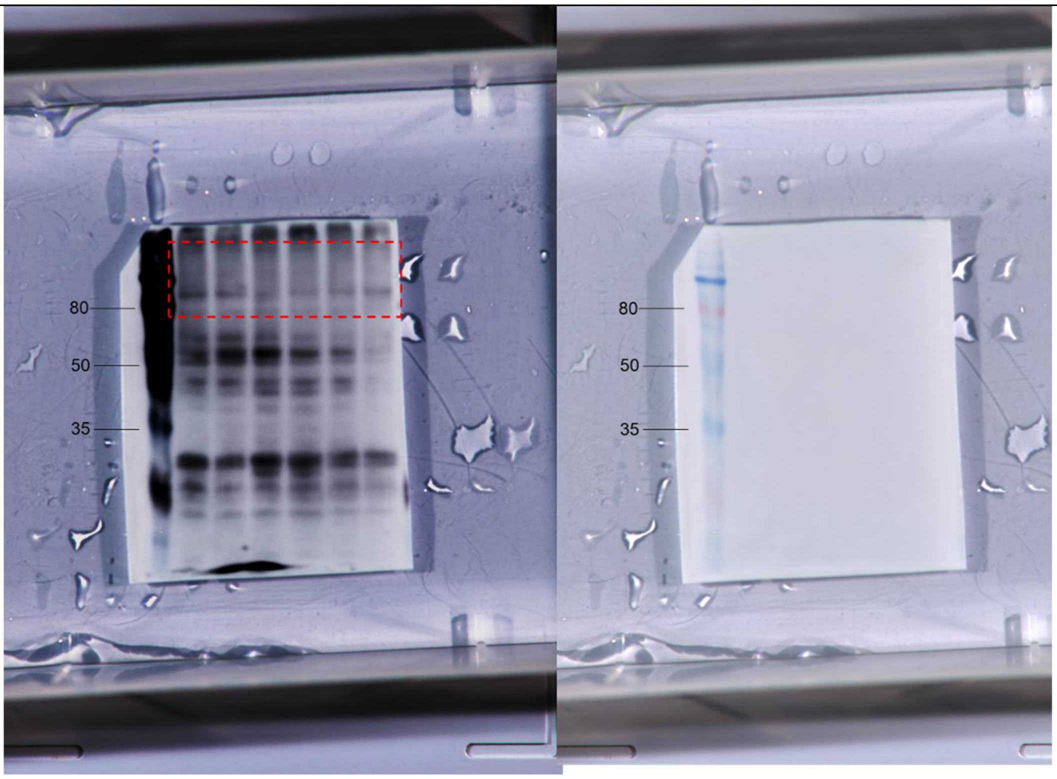

Figure S6.

SARS-CoV-2 NP

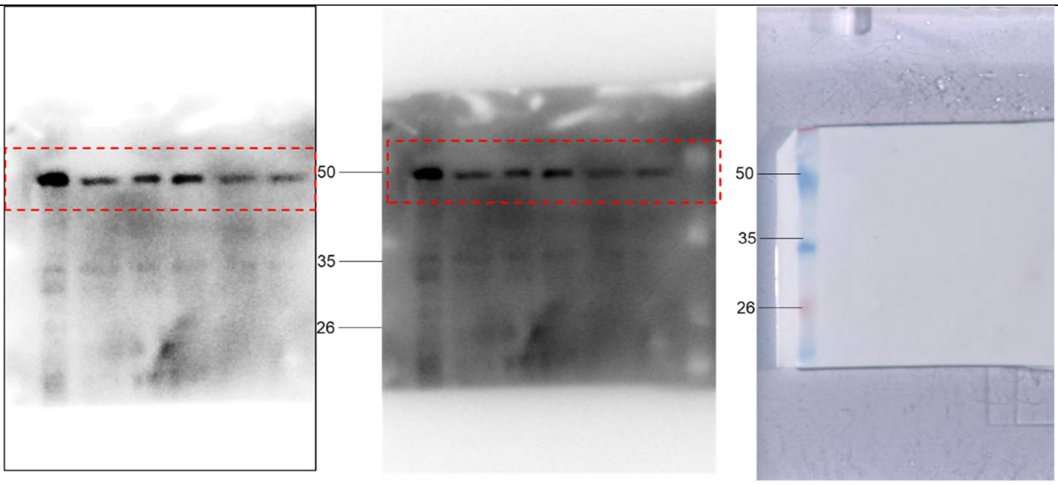

**Figure S7.**<br><br>GAPDH	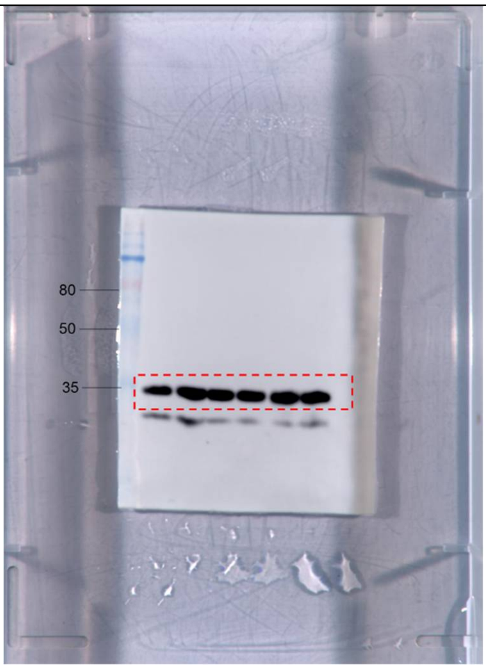
**Figure S8.**<br><br>IRF3	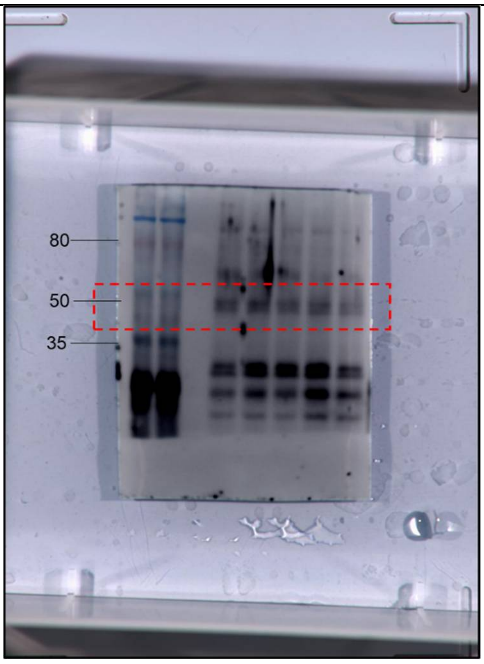

**Figure S9.** p-IRF3	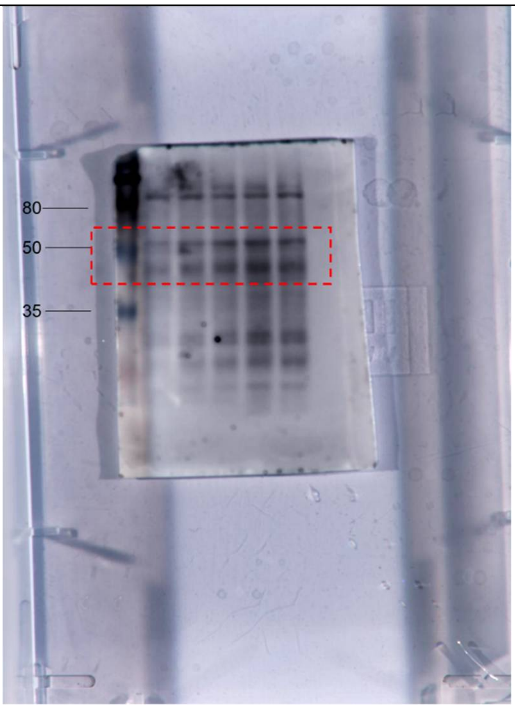
**Figure S10.** STAT1	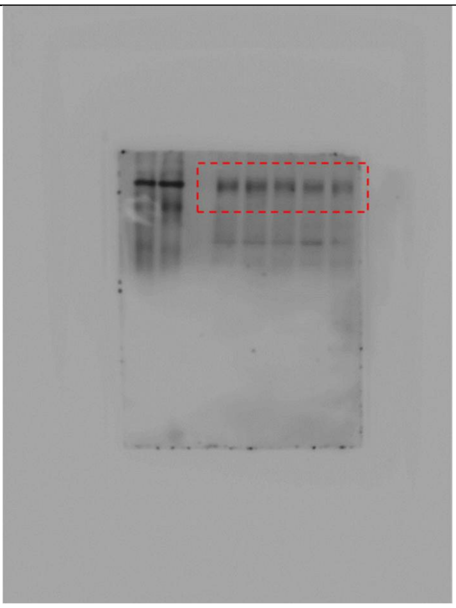 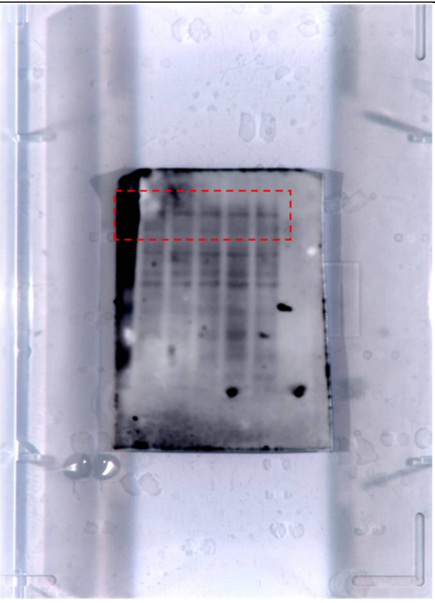

Figure S11.

p-STAT1

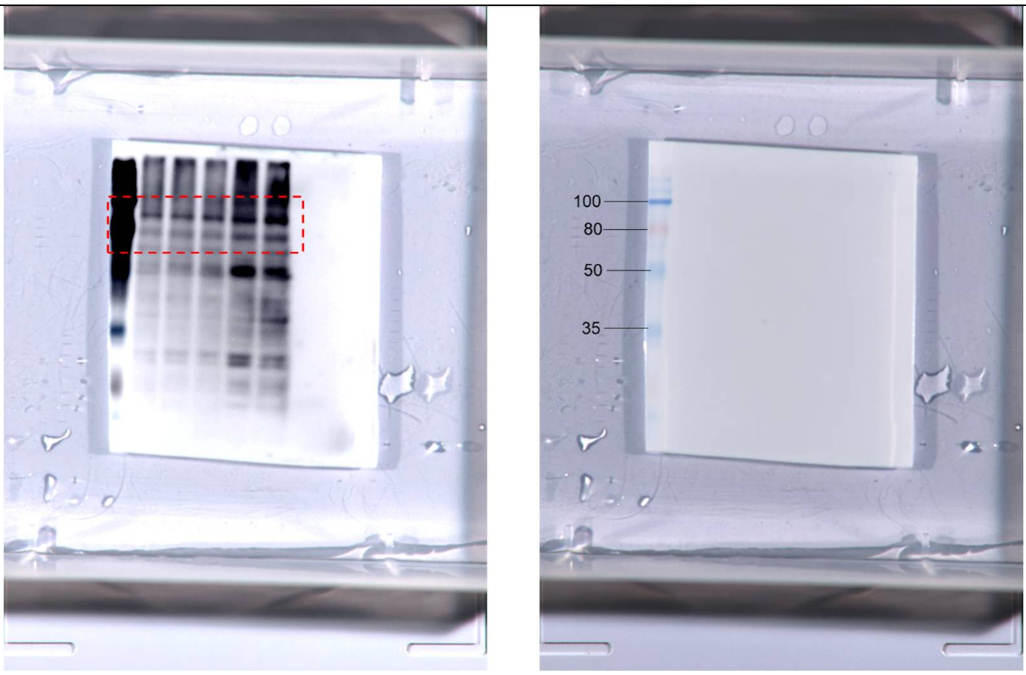

Figure S12.

SARS-CoV-2

NP

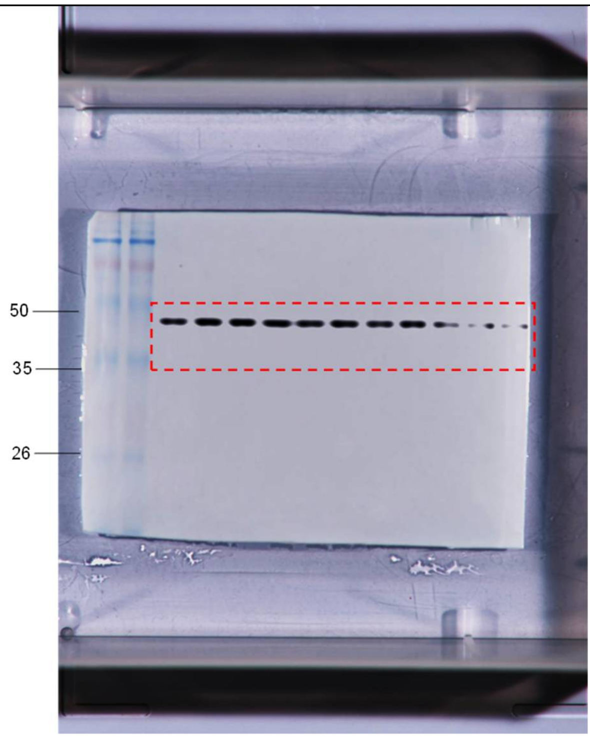

**Figure S13.**<br><br>GAPDH	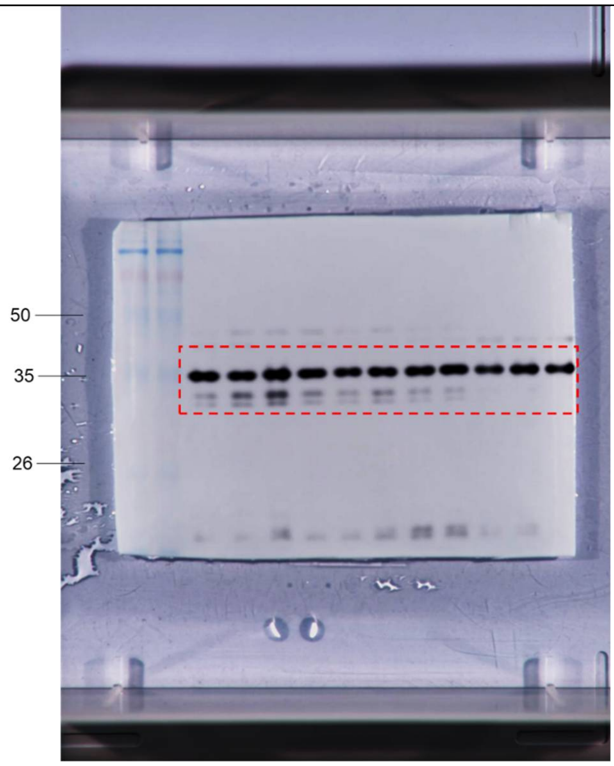
